# Fine-needle muscle microbiopsy: a feasible and well-tolerated alternative for skeletal muscle sampling

**DOI:** 10.3389/fphys.2026.1783535

**Published:** 2026-02-25

**Authors:** Johan Jakobsson, Karin Strigård, Apostolos Theos, Michael Svensson, Christer Malm

**Affiliations:** 1 Department of Community Medicine and Rehabilitation, Section for Sports Medicine, Umeå University, Umeå, Sweden; 2 Department of Community Medicine and Rehabilitation, Section for Physiotherapy, Umeå University, Umeå, Sweden; 3 School of Sport Sciences, Umeå University, Umeå, Sweden; 4 Surgery, Department of Diagnostics and Intervention, Umeå University, Umeå, Sweden

**Keywords:** fine-needle, minimally invasive, muscle biopsy, proteomics, skeletal muscle, tolerability

## Abstract

**Background:**

Conventional muscle biopsy techniques, such as the Bergström method, require large tissue samples and skin incisions. Fine-needle muscle microbiopsy offers a minimally invasive alternative, but data on tolerability are lacking. We aimed to present a refined minimally-invasive muscle microbiopsy protocol using a 20-gauge needle with topical anaesthesia and compare perceived pain with routine venipuncture.

**Methods:**

Twenty-six healthy adults (50% female) underwent vastus lateralis microbiopsy using a 20-gauge needle (0.9 mm). Pain was assessed immediately after the microbiopsy and a venous blood draw using a 21-gauge needle, with the visual analogue scale (VAS). Procedures were randomized.

**Results:**

Seventy-eight microbiopsies were successfully obtained. Mean pain scores were low for both procedures (microbiopsy: 1.0 ± 0.9; venipuncture: 1.4 ± 1.2) with no significant difference (P = 0.311). Most participants reported minimal or low discomfort (VAS ≤3) from the microbiopsy.

**Conclusion:**

Fine-needle muscle microbiopsy using a 20-gauge needle is well tolerated, with pain comparable to routine venipuncture. This approach substantially reduces invasiveness compared to traditional biopsies while providing adequate material for proteomic analysis. These findings support its ethical and practical application in sensitive populations and longitudinal research.

## Introduction

Muscle biopsy remains the gold standard for assessing molecular and structural properties of skeletal muscle, yet conventional techniques such as the current gold standard Bergström method (Bergstrom, 1962) require large tissue samples (≈100–250 mg) and involve skin incisions, limiting their use in vulnerable populations, including clinical cohorts, paediatric patients, and elite athletes who are reluctant to sacrifice substantial amounts of muscle tissue. Also, repeated sampling is highly suboptimal as the procedure itself will induce damage and inflammation (Malm et al., 2000). Fine-needle muscle microbiopsy has emerged as a minimally invasive alternative, enabling sampling with needles of 0.9–2.3 mm diameter and yielding 2–50 mg of tissue, which is sufficient for modern multi-omics workflows. State-of-the-art liquid chromatography mass spectrometry can identify thousands of proteins from a few micrograms of proteins (Murgia et al., 2021), reducing the need for large biopsies.

Despite its promise, most published microbiopsy protocols employ relatively large 13 to 16-gauge (G) needles (Hayot et al., 2005; Townsend et al., 2016; Pietrangelo et al., 2011), which may compromise tolerability and ethical acceptability in sensitive cohorts. Regulatory bodies, including the Swedish Ethical Review Authority, have requested empirical evidence comparing pain perception between muscle microbiopsy and routine venipuncture before approving studies in paediatric or clinical populations. Such data are currently lacking.

Here, we present a refined muscle microbiopsy protocol using a 20-G needle, smaller than those previously reported, and provide evidence of its clinical tolerability relative to standard venous blood sampling. This methodological advance reduces invasiveness while maintaining sample adequacy for downstream proteomic analysis, addressing a critical barrier to broader adoption in research and clinical settings.

## Methods

### Participants

Twenty-six healthy adults (13 men, 13 women; age 19–46 years) were recruited from a resistance training study (Jakobsson et al., 2021). Exclusion criteria included musculoskeletal injury, systemic disease, or cardiovascular/metabolic disorders. As we wanted a broad sample, participants exhibited a large age range and had different levels of physical activity. Most (n = 22), but not all, participants were physically active, of which 18 recreationally (1-3 session of exercise weekly, focusing on resistance training) while four participants performed structured exercise 3–6 times per week, mainly resistance training. Four participants were non-physically active and naïve to exercise, and no participants were highly competitive or elite athletes.

All participants provided written informed consent. The study was approved by the Swedish Ethical Review Authority (#2017-121-31) and was conducted in accordance with the Declaration of Helsinki.

### Biopsy procedure

**FIGURE 1:**
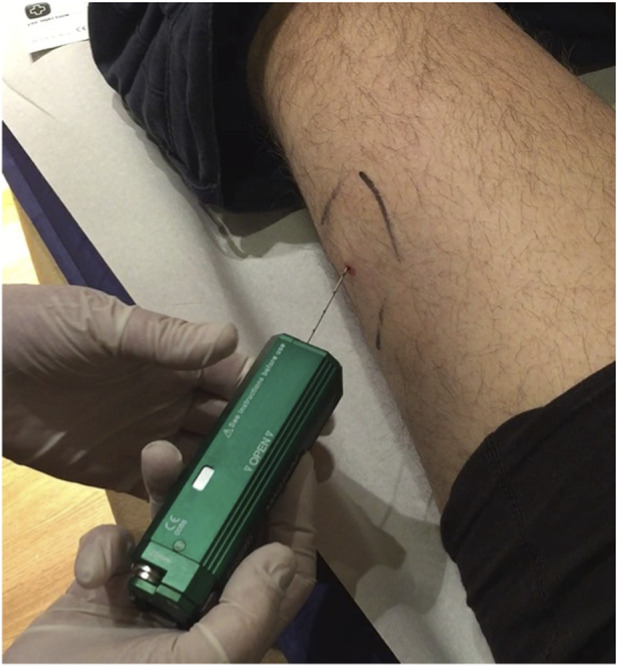
Microbiopsy of the vastus lateralis showing the 20-G needle fully inserted into the muscle.

Microbiopsies were obtained from the vastus lateralis using a 20-G automated spring-loaded biopsy needle (BARD Magnum®, GA, United States), as described by others (Figure 1) (Hayot et al., 2005). Local topical anaesthetic cream (Tapin, 2.5% lidocaine/prilocaine) was self-applied by participants according to instructions provided during the screening visit, 60–120 min prior to sampling. At baseline, three micro biopsies of approximately 2 mg each were collected at the midpoint between the lateral border of the patella and the anterior superior iliac spine, with sites spaced ∼1 cm apart. After the needle had passed through the fascia, it was advanced an additional 15 mm to ensure that the entire sample notch was positioned within the muscle.

Three microbiopsies were collected from the same limb to enable future assessment of inter-biopsy reliability in proteomic analyses (not reported herein). All three replicates were obtained within 10 min under identical conditions. Immediately upon extraction, each specimen was transferred into pre-chilled Eppendorf tubes for soft-tissue homogenization (PINKE1-RNA, Next Advance Inc., NY, United States) that had been placed on dry ice at the bedside and then moved to −80 °C storage. To preserve labile metabolites and minimize pre-analytical variation, we did not weigh the specimens at the point of collection and avoided any handling that would prolong time above cryogenic temperatures.

All microbiopsies were performed by KS (medical doctor, surgeon) and CM (professor of sports medicine). Prior to the study, they had completed approximately 5 and 15 pilot procedures, respectively, using the same device. KS had extensive experience with multiple skeletal muscle biopsy techniques, whereas CM had no previous hands-on biopsy experience. However, both operators achieved sufficient proficiency with this device after a limited number of pilot procedures. To ensure consistency, the two operators conducted calibration sessions to standardize all steps of the microbiopsy procedure. Venous blood draws were performed by a registered nurse experienced in phlebotomy.

Immediately after the first microbiopsy at baseline, participants were asked to evaluate perceived pain using a non-hatched 100 mm visual analogue scale (VAS) (Bijur et al., 2001) anchored with “no pain” and “worst imaginable pain.” Participants also rated pain from a venous blood draw performed with a 21-G needle (BD Vacutainer® Eclipse™ Signal™), without local anaesthetics. The order of procedures was randomized using simple randomization. Pain ratings were collected immediately after the first microbiopsy to capture acute pain perception attributable to a single needle insertion and firing event, minimizing confounding from cumulative discomfort, local sensitization, or anticipatory effects that could arise across repeated samplings within a short period. Because the primary aim was to benchmark tolerability to a single microbiopsy procedure against venipuncture, the first sampling event was considered the most directly comparable.

Pain ratings were compared using descriptive statistics and a Wilcoxon signed-rank test (JMP Pro, v.18). Statistical significance was set at α = 0.05.

## Results

A total of 78 microbiopsies were successfully obtained. Pain ratings for the first microbiopsy and venipuncture are summarized in Table 1. Each microbiopsy yielded approximately 2 mg of skeletal muscle tissue.

**TABLE 1:** Pain ratings during micro biopsy and venepuncture.

Sampling method (N = 26)	Pain score		
	Mean ± SD	Median (IQR)	Min – Max
Microbiopsy	1.0 ± 0.9	1 (0–2)	0–3
Blood sampling	1.4 ± 1.2	1 (1–2)	0–5

There was no significant difference in pain between procedures (Z(25) = 1.01, P = 0.311). All participants (100%) reported low pain (VAS ≤3) for microbiopsy, compared to 83% for venepuncture. No adverse events occurred during or following the microbiopsy procedure. While participants were not systematically asked to record pain or discomfort at 24–72 h post-procedure, they were instructed to contact the study team in case of delayed pain, bleeding, hematoma, or other adverse events. All participants initiated supervised whole-body resistance training within a few days after the micro biopsy, and no participant reported post-biopsy pain or discomfort.

## Discussion

The present feasibility study demonstrates that fine-needle muscle microbiopsy of the vastus lateralis using a 20G needle in healthy adults is both feasible and clinically tolerable.

With only a self-applied topical anesthetic cream, most participants reported minimal or no pain, and ratings were comparable to or lower than routine venipuncture. However, we should note that local anaesthetic cream was applied before the microbiopsy, whereas venous blood sampling was performed without analgesia, as is standard practice. This difference may influence direct pain comparisons. Also, the venipuncture used a 21-G needle, which has a smaller outer diameter than the 20-G needle used for the microbiopsy, and therefore may inherently cause less discomfort. Future studies should consider standardized analgesia or include a no-anaesthetic microbiopsy condition to isolate intrinsic pain levels.

Although not directly examined in the present study, reported pain levels are markedly lower than those typically reported for standard Bergström biopsies, which require injection of local anesthetic and are commonly rated in the 4–5 range on a 0–10 scale (Hayot et al., 2005; Dengler et al., 2014). Beyond improved tolerability, the fine-needle approach substantially reduces tissue burden while providing sufficient material (1–2 mg) w.w. muscle tissue, which is well enough for advanced multi-omics analyses using tandem mass spectrometry. Yet, it should be noted that using this small of a biopsy needle do not provide sufficient material for traditional immunohistochemistry methods, including fibre-type composition analyses. However, this is possible using mass-spectrometry, which requires only microgram quantities of protein (Murgia et al., 2021).

A limitation in this study is that delayed soreness or discomfort at (i.e. 24–72 h) was not systematically recorded. While no delayed adverse events were reported to the research team, the absence of structured follow-up limits conclusions regarding short-term post-procedural soreness or localized inflammatory responses. Future studies should include standardized follow-up measures. Procedural success, complication rates, and perceived pain may be influenced by operator experience with both microbiopsy and venipuncture. In the present study, procedures were performed by trained personnel; however, we did not quantify the effect of operator expertise on pain outcomes. Further, one should consider further standardization (e.g., insertion depth, and where feasible ultrasound guidance) to minimize variability and enhance safety, which may be particularly pertinent in paediatric cohorts.

We did not record tissue mass at the time of sampling because the protocol prioritized rapid cryopreservation to preserve labile metabolites. As a result, we cannot report intra-subject variance in tissue yield across replicates in this manuscript. Nevertheless, on the basis of core dimensions and prior protocol experience, each biopsy provided >1 mg of tissue as documented in pilot biopsies, which is well above the minimum input needed for modern proteomics such as UHPLC-LC/MS-MS.

These findings address a key ethical and practical concern for applying muscle biopsy in vulnerable populations and support integration of this technique into longitudinal and interventional research.

Future work should formally quantify biopsy mass and protein content, establish reproducibility regarding protein- and gene-expression, sample integrity for multi-omics applications, and feasibility in paediatric and clinical cohorts. By combining minimal invasiveness with good tolerability, the 20G fine needle microbiopsy shows potential for broader research and clinical application.

## Data Availability

The raw data supporting the conclusions of this article will be made available by the authors, without undue reservation.
